# HDL-Chitosan Nanoparticles for siRNA Delivery as an SR-B1 Receptor Targeted System

**DOI:** 10.2174/1386207326666230406124524

**Published:** 2023-08-25

**Authors:** Rasim Masimov, Gülay Büyükköroğlu

**Affiliations:** 1 College of Pharmacy and Nutrition, University of Saskatchewan, Saskatchewan, Canada;; 2 Department of Pharmaceutical Biotechnology, Faculty of Pharmacy, Anadolu University, Eskisehir, Turkiye

**Keywords:** HDL, SR-B1 receptor and ApoA-1, silencing gene-Bcl-2 siRNA, chitosan nanoparticles, antisense technology-gene delivery, targeting therapy-liver cancer

## Abstract

**Aims:**

High-Density Lipoprotein (HDL) is a complex structure unique to the human body. ApoA-1 protein is a significant structural/functional protein of HDL and provides a natural interaction with the SR-B1 receptors on the cell membrane. The overexpression of the SR-B1 receptor in the membrane of malignant cells suggests that targeting cancer cells can be possible using HDL. The objective of this study was to prepare HDL-conjugated chitosan nanoparticles containing a genetic material that can be used for liver cancer.

**Methods:**

HDL used in the preparation of the formulations have been obtained by isolating from blood samples taken from healthy volunteers. Bcl-2 siRNA inhibiting BCL-2 oncogene was selected as the genetic material. Chitosan nanoparticles were prepared using the ionic gelation method utilizing low molecular weight chitosan. Physicochemical properties of formulations, transfection efficacy, and cytotoxicity of them on 3T3 and HepG2 cell lines were examined.

**Results:**

The average diameters of the selected formulations were below 250 nm with a positive zeta potential value between +36 ± 0.1 and +34 ± 0.5 mV. All formulations protected Bcl-2 siRNA from enzymatic degradation in the presence of serum. Cellular uptake ratios of particles by HepG2 cells were found to be between 76% and 98%. HDL/chitosan nanoparticles/Bcl-2 siRNA complex was found to be more toxic when compared to chitosan nanoparticles/Bcl-2 siRNA complex and naked Bcl-2 siRNA.

**Conclusion:**

According to attained results, the HDL-conjugated chitosan nanoparticles can bring advantages for targeted siRNA delivery to malignant cells that overexpress SR-B1 receptors, such as HepG2.

## 1 INTRODUCTION

The use of siRNA in gene therapy for cancer is an area of great interest today. It has been pointed out that using siRNA in treatment is more effective and harmless than conventional drugs [1]. The therapeutic use of siRNA is generally based on the mechanism known as Antisense Technology of silencing the desired gene using siRNA oligonucleotides. This usage is one of the methods of antisense technology in which synthetic nucleic acids are used to regulate gene expression in cells [2, 3]. There are many studies conducted with siRNA for cancer treatment, aiming to silence the genes that cause cancer [4]. Carrying small oligonucleotides such as siRNA to cancer cells causes the cell's death by silencing the gene and also increases the sensitivity of cells that have become resistant to chemotherapy and radiotherapy to these treatments [5-8]. One of the crucial genes to be silenced to prevent cancer development is the Bcl-2 gene. The overexpression of Bcl-2 is seen in some cancer types and has a vital role in cancer development; therefore, silencing this gene is essential for cancer treatment [9]. Radiotherapy is used for the apoptosis of cancerous cells in cancer treatment. However, recent studies have revealed that radiotherapy is sometimes insufficient for cell apoptosis. The blocking of cell apoptosis by the Bcl-2 gene explains the reason for this deficiency. In other words, overexpressing the Bcl-2 gene increases resistance to radiotherapy [10]. Also, silencing the Bcl-2 gene is crucial for the effectiveness of chemotherapy. Chemotherapy resistance, which develops in cancer cells over time and, depending on the dose, reduces the apoptosis effect of chemotherapy on the cell. It has been reported that the Bcl-2 gene needs to be silenced for better results in chemotherapy [11]. Since the naked siRNA is digested by enzymes in the blood or captured by the reticuloendothelial system (RES), it is degraded before reaching the targeted organ/cell [12]. Lots of carrier systems have been developed to overcome these handicaps. Recent studies on using HDL (High-Density Lipoprotein) nanoparticles as a gene delivery system have shown that they are safe and efficient vectors for transporting genetic material to specific cells/organs by overcoming the abovementioned obstacles [13]. Cholesterol-conjugated siRNA was able to silence gene expression after *in vivo* application. Conjugation of siRNA to cholesterol, bile acids, and long-chain fatty acids mediates siRNA transfection into cells and induces the targeted gene to be silenced. The efficient and selective uptake of such siRNA conjugates depends on the interaction of conjugates' lipoprotein particles, lipoprotein receptors, and transmembrane proteins [14]. M. Feng *et al.* showed that HDL nanoparticles are more effective than other delivery systems for drug targeting, especially in the liver [15]. HDLs are endogenous nanoparticles consisting of a hydrophobic core and a hydrophilic outer layer. HDL's core consists of cholesterol esters and triglycerides, while its outer layer contains apolipoproteins and lipids [16, 17]. The biosynthesis of HDL in the body begins with the synthesis of ApoA-1 (Apolipoprotein AI) in the liver and intestines. After that, released lipids and cholesterol *via* ATP-ABCA1 (binding cassette transporter AI) receptors combine with this lipid-free ApoA-1 to form HDL nanoparticles [18]. The mission of these formed nanoparticles is to ensure the transport of various endogenous molecules such as protein, vitamins, hormones, and miRNAs in the blood to different organs and to allow them to be metabolized. HDL particles can involve reverse cholesterol transport (RCT) as the primary carrier for delivering cellular cholesterol to the liver [13]. Circulating HDL particles are taken into cells mainly *via* scavenger receptor class B type 1 (SR-B1) receptors overexpressed in the liver and various malignant cells. ApoA-1 protein, which can be bound to the SR-B1 receptor, is a structural and functional HDL protein [16]. Multiple studies have shown that HDL can be advantageous in cancer treatment due to its binding to the SR-B1 receptor *via* ApoA-1 [19, 20]. Chitosan has cationic, biocompatible, biodegradable, non-toxic, inexpensive, and low immunogenic properties. Thus, it is a widely used polymer as a gene delivery system [21]. HDL was conjugated with chitosan to load active substances and to use the bioadhesive feature of chitosan due to surface charge and increased HDL stability. The prepared HDL/chitosan nanoparticle evaluation determined that the active substance's bioavailability was increased, and HDL's stability was preserved [22]. Chitosan is also one of the most studied polymers in non-viral gene therapy. Their positive charge under slightly acidic conditions allows interaction with siRNA and the formation of complexes or nanoparticles [23]. However, it needs further development to mediate high gene silencing, especially after intravenous injection [24]. Within the scope of our study, to deliver Bcl-2 siRNA to liver cancer cells more stably *via* the SR-B1 receptor, HDL/chitosan nanoparticles were prepared. This non-toxic and highly bioavailable siRNA delivery system was characterized, and its efficacy on HepG2 liver cancer cells was investigated *in vitro*.

## 2 MATERIALS AND METHODS

### 2.1 Materials

First, HDL was isolated from blood samples taken from volunteers to prepare HDL-conjugated chitosan nanoparticles. HDL used in the preparation of the formulations has been obtained by isolating from approximately 10 mL of blood samples taken from each of 41 healthy and drug-free volunteers aged 20-50 years, by Decision No:29 with Approval Date 06.02.2018 of the Ethics Committee of Non-Interventional Clinical Researches of Eskişehir Osmangazi University. An 'Informed Consent Form,' approved by the Ethics Committee, was obtained from each volunteer participating in the study. Low molecular weight chitosan and Tripolyphosphate (TPP) were obtained from Sigma-Aldrich (Germany). siRNA Bcl-2 was selected as genetic material, and Fluorescein (FITC) Conjugate siRNA used for transfection studies was obtained from Santa Cruz Biotechnology (USA). Spectra™ Multicolor Broad Range Protein Ladder used as protein marker in SDS-PAGE was obtained from Thermo Scientific (USA), and STA-607 Ultracentrifuge-free HDL Purification kit, used for HDL isolation, was obtained from Cell Biolabs (USA). The Dimension Flex Reagent AHDL-C kit was obtained for HDL quantification from Siemens (Germany).

### 2.2 Obtaining HDL Cholesterol and ApoA-1 Protein

#### 2.2.1 HDL Isolation

The method used by Wiebe and Smith (1985) was chosen to obtain HDL. The blood samples taken from the volunteers were kept at room temperature for 30-60 minutes until they were coagulated and then centrifuged for 30 minutes at 1,500xg at 20°C. The supernatant portions (serum) were collected in 50 mL plastic test tubes and kept at -20°C until HDL isolation [25].

HDL Purification Kit (Ultracentrifugation Free) (STA-607; Cell Biolabs, USA) was used to isolate HDL from Serum. The procedure for the kit used (htpp-1) is given below. While HDL isolation was performed using the kit, 24 mL of serum was used each time. The serum stored at -20°C was thawed at room temperature and transferred to 2 mL microtubes as a 1 mL sample in portions. 5 µl of Dextran solution and 50 µl of precipitation solution were added to the samples and incubated on ice for 5 minutes by mixing with the help of a pipette. After incubation, it was centrifuged at 6,000xg at +4°C for 10 minutes, and the supernatant was transferred to new tubes. 60 µL of Dextran solution and 150 µL of precipitation solution were added and incubated for 2 hours at room temperature. After incubation, the samples were centrifuged at 18.000-20.000xg at +4°C for 30 minutes, and the supernatants were discarded. 500 µL HDL Resuspension Buffer was added to the pellet and mixed with the help of a pipette. After the resuspended mixture was centrifuged under 6000xg at +4°C for 10 minutes, the supernatant was discarded. 600 µL of 1X HDL washing solution was added to the pellet, and the tubes were shaken for 30 minutes at 4°C at 160 rpm by paying attention to the formation of foaming. After shaking, the samples were centrifuged at 6,000xg at +4°C for 10 minutes, and the supernatants were taken into different tubes. 90 μL of the kit solution was added, pipetted, and incubated at +4°C for 1 hour to remove the dextran in the supernatant. Then, it was centrifuged under the same conditions. HDL-containing supernatants were collected in 15 mL plastic test tubes, and some HDL-containing solution to be used in the short term was separated and stored at +4°C and the rest at -20°C.

#### 2.2.2 HDL Quantification

The Dimension^®^ RxL Max^®^ integrated chemistry system (Siemens) and Dimension Flex Reagent AHDL-C kit were used to determine the concentration of the HDL solutions. For this, 3 µL HDL containing supernatant was placed in each of the 3 sample chambers of the device. After a reaction time of 8.6 minutes at 37 ± 0.1°C, automatic measurements were carried out at 600 and 700 nm wavelengths with the biochromatic endpoint method. HDL quantification was performed by taking the average of the measurements obtained.

#### 2.2.3 Determination of the Presence of the ApoA-1 Protein

The SDS-PAGE method was used to determine whether the isolated HDL is pure, to determine the location of the ApoA-1 protein in the gel, and to isolate it from this region. The SDS-PAGE method was performed according to the procedure described by Brace (2010) with some modifications. For this, two different gels of 10%-polyacrylamide with 1.5 mm diameter and ten wells were prepared using the Protean III Mini system (Bio-Rad Laboratories, US), and the samples were loaded into these gels [26].

HDL samples were loaded onto gels after taking 7.5 µL of HDL solution, adding 7.5 µL of loading buffer to it, and leaving it in a water bath at approximately 90°C for 3 minutes. 10 µL of the colored protein marker sample used to determine the protein's location was taken, and 10 µL of loading buffer was added to it and loaded to the gel. Electrophoresis was first performed at 80 V and 25 mA for 30 minutes and then at 120 V 40 mA for 80 minutes. At the end of electrophoresis, one of the gels was washed twice with distilled water and left in a shaker with 40 mL of fixation solution (10% Glacial acetic acid, 50% Methanol, 40% distilled water) for 1 hour. After the fixation process, the fixation solution in the container was taken, and the dyeing solution (0.1% Coomassie Brilliant Blue, 40% ethanol, 10% acetic acid) was poured into the container. The container was shaken on a shaker for about 20-30 minutes. Finally, destain solution (40% ethanol, 10% acetic acid) was added to remove the staining solution and left in the shaker until the dye was completely removed from the non-protein areas. This stained gel was used to locate the ApoA-1 protein.

#### 2.2.4 Isolation of the ApoA-1 Protein

At the end of the electrophoresis, the second gel was removed, transferred to the container, and washed with distilled water. This unstained gel was placed on the stained gel and cut horizontally as a strip from the colored band line corresponding to the molecular weight of the ApoA-1 protein. The cut gel strip was cut into 3-4 mm squares and transferred to 15 mL tubes containing 4 mL of distilled water and left to agitate overnight. The next day, the tubes were removed from the shaker, centrifuged to precipitate the gel fragments, and the liquid portion (protein-containing solution) was removed and transferred to separate tubes. In order to make the protein solution more concentrated, it was moved to 5-10 kDa protein concentrator (vivaspin) columns with a volume of 500 µL and centrifuged. The concentrated protein solution was stored at -20°C until use.

### 2.3 Preparation of Chitosan Nanoparticles

The ionotropic gelation method was used to prepare chitosan nanoparticles. The mechanism of this method is based on the electrostatic interaction between the amine group of chitosan and the negatively charged polyanions such as TPP [27]. For this purpose, firstly, chitosan was dissolved in %2 acetic acids to prepare a % 0.5 (w/v) chitosan solution, and its pH was adjusted to 5 with a 2M NaOH solution. Finally, TPP, an aqueous solution at a concentration of 0.7mg/mL, was added dropwise to the chitosan solution stirred at 400 rpm with a weight ratio of chitosan: TPP of 4:1. The nanoparticle suspensions were gently stirred using magnetic stirring for 30 min at room temperature before being subjected to further analysis and applications.

#### 2.3.1 Preparation of HDL/Chitosan Nanoparticles

Also, HDL/Chitosan nanoparticles were prepared using the same method. For this purpose, TPP was mixed with 500µL, 250 µL, 100 µL, and 75 µL of HDL solutions (5.7mg/mL) and then added to 2.24 mL 0.5% chitosan solution as described before.

#### 2.3.2 Preparation of Nanoparticles-siRNA Complexes

The adsorption method, which is frequently used in gene delivery systems, was used to prepare nanoparticle-siRNA complexes. Complex formation between positively charged nanoparticles and negatively charged genes occurs *via* electrostatic interactions [23]. For adsorption of siRNA to both chitosan and HDL/Chitosan nanoparticles, 8μl, 10μl, 12μl, 15μl, and 20μl formulations were taken into different microtubes. 10μl Bcl-2 siRNA (20μg / mL) was added to each and mixed gently using a micropipette. For electrostatic interaction between nanoparticles and siRNA, tubes were left to incubate for 30 minutes at room temperature. Fig. (**1**) shows the preparation of HDL/Chitosan/siRNA of formulations.

### 2.4 Characterization Studies of Formulations

#### 2.4.1 Investigation of the Surface Morphology of the Particles

The examination of the surface morphology was performed with the scanning electron microscope (SEM) (Zeiss Ultra Fesem, Germany). For this, the formulation was first diluted with distilled water, dripped onto the double-sided carbon tape attached sample stub, and left to dry at room temperature. Finally, the samples were coated with a thin gold layer (100Å) and visualized by SEM at high vacuum and 8kV acceleration voltage.

#### 2.4.2 Particle Size, Polydispersity (PDI), and Zeta Potential

Formulations were characterized using a particle size analyzer (Zetasizer NanoZS, Malvern Instruments, UK) to determine the average diameter and size distribution represented by the polydispersity index (PDI). The samples were prepared by adding 0.22 µm filtered distilled water with 950 µL of pH 7.4 and 50 µS conductivity to the 50 µL formulation. For particle size and PDI, 10 measurements were taken for each sample, and the average value was automatically found after three repeats.

The same samples were used to determine the zeta potentials, 20 measurements were made, and this process was repeated three times. The Helmholtz-Smoluchowski equation was used to convert the electrostatic mobility into zeta potential.

#### 2.4.3 Gel Retardation Studies

The agarose gel electrophoresis method was used to determine the binding rates of the genetic material loaded in the formulations and the possible change of these rates in the presence of serum. For this purpose, a 2% agarose gel containing ethidium bromide was prepared. Samples adsorbed at specific rates of siRNA were loaded on this gel, and electrophoresis was performed using Tris/Borate/EDTA (TBE) buffer at 50 mV for ~2 hours. Images were obtained using a gel documentation system (Uvitec Alliance 4.7, Cambridge, UK).

The behavior of the siRNA-loaded formulations in the medium containing serum components was investigated to determine the ability of the prepared formulations to protect the genetic material against serum components. For this process, formulations loaded with 200 ng siRNA at the rate used during transfection were incubated with DMEM containing 10% FBS at 37°C. At the end of 1, 4, 10, 24, and 48 hours, samples were taken, loaded on agarose gel, and visualized.

### 2.5 Cell Culture Studies

HepG2 (human liver cancer cell line), and 3T3 (healthy mouse fibroblast cell line) were used to determine the efficacy of the prepared formulations. Cells were cultured in a DMEM medium containing 10% FBS, 1% penicillin-streptomycin, and 1% L-glutamine and incubated at 37°C in atmospheric conditions containing 5% CO_2_ and 95% air.

The colorimetric 3-(4,5-dimethylthiazol-2-yl)-2,5-diphenyltetrazolium bromide (MTT) test developed by Mosmann was used to determine the cytotoxicity of formulations and the efficacy of genetic material *in vitro* [28]. The experimental setup was arranged and carried out as in our previous study [29]. The experiment was performed in 8 wells for each concentration of the formulations and repeated three times.

#### 2.5.1 Transfection Studies

Transfection studies were performed by adsorbing FITC-conjugated control siRNA to the formulations instead of Bcl-2 siRNA to determine the ability of the formulations to transfer genetic material into the cell using the same method in our previous study [4]. After adding 200 ng FITC-siRNA to 10 µL formulations, it was incubated at 37°C for 30 minutes for adsorption. These transfection studies were performed in HepG2 and 3T3 cell lines. Phase contrast and fluorescence images of cells were taken by a fluorescence microscope (Leica Dmls, Switzerland) with an objective lens of 20x magnification [29]. Transfection rates were calculated using the following equation after counting cells with and without transfection was performed automatically on a plate reader (BIOTEK Brand Cytation5 Model, USA).

TE(%) = NTC / NTC + NnTC *x* 100

TE: Transfection Efficiency

NTC: Number of transfected cells

NnTC: Number of non-transfected cells

#### 2.5.2 Statistical Analysis

For cell culture experiments, statistical data analysis was performed using one-way analysis of variance (ANOVA) and regression analyses. The data are represented as means and standard deviations. Differences were considered significant at *p* < 0.05 to be statistically significant in all cases.

## 3 RESULTS AND DISCUSSION

### 3.1 Isolation and Quantification of HDL

HDL, also known as good cholesterol, helps remove other cholesterol forms from your bloodstream. Two standard methods are used for the isolation of lipoproteins from the blood. One is the precipitation method, based on the interaction between the negatively charged groups on the polyanions and the positively charged groups on the protein parts of the lipoproteins. At the same time the other is the ultracentrifugation method, based on the density of the lipoproteins [30]. Our study used a commercial kit to isolate HDL from blood (HTTP-1). This method facilitated the recovery of pure HDL from the blood by selective precipitation of HDL with Dextran Sulphate without the need for ultracentrifugation. After the pure HDL solutions were collected in a single stock, the HDL concentration was determined as 5.7 mg/mL according to the measurement in this stock solution. HDL in the blood plays a vital role in promoting the efflux of excess cholesterol from the tissues to the liver. ApoA-1 protein, approximately 70% of HDL’s total protein, is a significant structural and functional protein for HDL. It is thought that ApoA-1 is to be present in almost all HDL particles. ApoA-1 is a protein that can bind strongly to lipids and possesses detergent-like solid properties. The main functions of ApoA-1 are to interact with cellular receptors, activate lecithin/cholesterol acyltransferase (LCAT), and provide multiple antiatherogenic activities to HDL [16]. In these ways, it provides cholesterol loading from tissues to HDL as a cargo. It then binds to the SR-B1 for this cargo to be poured into the liver. ApoA-1 also performs receptor-mediated endocytosis.

It was determined that the SR-B1 receptor is overexpressed in malignant cell lines such as breast, prostate, ovarian, pancreatic, nasopharyngeal, and colorectal cancers. This situation increases the cellular uptake of more cholesterol through SR-B1, and accordingly, the rapid proliferation of the cells is promoted [16, 31]. With the ApoA-1 isolation, this study aims to prove whether HDL is isolated in a pure form and whether ApoA-1 protein exists. Since our primary goal is the efficient delivery of siRNA by targeting HDL-conjugated chitosan nanoparticles to liver cancer cells using the interaction between ApoA-1 and SR-B1 receptor, the isolation of HDL and determination of the presence of ApoA-1 on it is critical. The SDS-PAGE gel electrophoresis method was used to determine the presence of ApoA-1 in HDL solution. ApoA-1 is a protein with a molecular weight of 28.3 kDa [32]. Thus, its band was expected to locate between marker protein bands at 25 kDa and 35 kDa on the gel. As seen in Fig. (**2**), a band was observed in the mentioned area on Coomassie blue stained gel. As expected and as observed in other studies [26, 32], it was determined that ApoA-1 was observed in higher amounts in the gel than in other protein structures.

The presence of ApoA-1 was also demonstrated by the second SDS-PAGE analysis presented in Fig. (**2**), which was performed by removing the 25 kDa to 35 kDa protein band areas on the gel in Fig. (**2**). These results indicate that the ApoA-1 protein, hence HDL, was successfully isolated and purified from blood.

### 3.2 Features of Nanoparticles

Chitosan nanoparticles can be prepared using different methods such as ionic gelation, polyelectrolyte complex (pec), precipitation, complex coacervation, microemulsion, emulsifying solvent diffusion, and covalent crosslinking [33-35]. Our study used the ionic gelation method to prepare the chitosan nanoparticles. The ionic gelation method is the most widely used method for preparing chitosan nanoparticles as genetic material carrier systems.

This method is preferred for the formulation of biological products due to its advantages, such as having aqueous processing conditions, using non-toxic reagents, requiring low energy, and not requiring harsh mechanical conditions [36]. The concentrations and ratios of chitosan and TPP, the molecular weight of chitosan, and the pH value of the chitosan solution are the factors that can affect the size and surface charge of the nanoparticles prepared by ionic gelation. It has been reported that the small particle size of nanoparticles prepared using low molecular weight chitosan depends on the chitosan/TPP mass ratio [27, 36, 37]. As a result of our experiments, considering the particle size and zeta potential of the nanoparticles, it was found that the 4/1 ratio for chitosan/TPP was the most appropriate rate. 2.24 mL chitosan solution (0.5%) adjusted to pH five was used to prepare all formulations.

#### 3.2.1 Particle Size, Polydispersity (PDI), and Zeta Potential

Particle size and size distribution are among the most critical features of drug delivery systems. These properties determine the *in vivo* distribution, biological behavior, toxicity, and targeting ability of delivery systems [27, 36, 37]. It has been reported by Al-Nemrawi *et al*. that the particle size and dispersion are reduced by increasing the mixing speed from 500 rpm to 1,500 rpm [38]. Since genetic material and protein structure will be used in our study, the formulations have been optimized to obtain small particle sizes at the lowest possible rpm, considering the possibility of high-speed mixing to damage these materials. As a result of studies on the mixing speed, the chitosan dispersion with the smallest particle size and PDI, of approximately 170 ± 10 nm and 0.285 ± 0.05, was formed using a 400-rpm mixing speed without the need for high-speed mixing. This dispersion, coded as K3, was used as the primary formulation for HDL, siRNA, and HDL/siRNA-loaded formulations. Table **1** presents formulations' codes, particle size, and PDI values.

It was determined that the particle size and PDI value increased after HDL conjugation to the K3 formulation and this increase was dependent on the amount of HDL (Table **1**).

The sizes of HDL/Chitosan nanoparticles that coded as K3-H1, K3-H2, K3-H3, and K3-H4 were found at 625 ± 75nm, 460 ± 17nm, 376 ± 26nm, 240 ± 10nm. They correspond to 500µL, 250 µL, 100 µL, and 75 µL of HDL volumes, respectively. This finding was interpreted as the excess HDL adsorbed on the cationic HDL/chitosan nanoparticles’ surface by electrostatic interaction. The adsorbed HDL in this complex may have interacted electrostatically with other cationic HDL/Chitosan nanoparticles and caused the aggregation of particles [39].

Particle size is an essential factor for the cellular uptake of nanoparticles. In addition, cell types have differences in the cellular uptake of nanoparticles. For example, using clathrin or caveolin-mediated endocytosis, the cell takes into nanoparticles in the 120-150 nm size range. Nanoparticles in the 250 nm to 3 µm size range are taken into the cell by *in vitro* phagocytosis. At the caveolae-mediated pathway, the caveolae size inhibits the uptake of larger nanoparticles. Thus, multiple endocytic pathways are used for cellular uptake, depending on the size of the particles [40]. The particle size of the K3 and K3-H4 formulations was found at 170 ± 10 - 240 ± 10 nm. This result indicates that these two formulations are suitable for cellular uptake since their sizes are below 250 nm, except for the possibility of SR-B1 receptor interaction with the ApoA-1 protein on the HDL surface.

The PDI is a ratio in the range of 0-1 that gives information about the homogeneous distribution of particle size in a dispersion system. The decrease in the ratio toward zero indicates that the dispersion system achieves the highest dispersion quality with a uniform and homogeneous distribution of the particles. Although optimum values for PDI are accepted as ≤0.3, most researchers also accept values of ≤ 0.5. Therefore, it is reported that PDI should be less than ≤ 0.5 for polydisperse systems [41, 42]. Since the PDI values of the K3 and K3-H4 formulations were determined as 0.285 ± 0.05 and 0.251 ± 0.05, it was concluded both formulations had optimum polydispersity.

Zeta potential is an essential physicochemical parameter. It affects the stability of nanosuspensions, cellular uptake of nanoparticles, and the loading of drugs or genetic materials [43]. It has been reported that nanoparticles with cationic properties provide strong electrostatic interactions with negatively charged phosphate groups of the cell membrane, which creates pores on the cell membrane due to surface tension. These pores allow nanoparticles to be taken into the cell by the non-endocytosis mechanism [40].

The zeta potential of nanoparticles is also vital to facilitating the loading of genetic materials. The genetic material has a negative charge as it contains phosphate groups. It can be adsorbed on the particle surface or encapsulated by the system through electrostatic interaction with the cationic carrier system materials [43]. It was determined that all formulations in our study had positive zeta potential values in the range of 36 ± 0.1 and 27 ± 1.0 (Table **1**). But, the K3-H4 formulation was chosen as the siRNA carrier system due to its small particle size (240 ± 10 nm), low PDI value (0.25 ± 0.05), and high zeta potential (34 ± 0.5). And further experiments were carried out in this formulation. K3-H4 formulation was chosen as the siRNA carrier system due to its small particle size (240 ± 10 nm), and low PDI value (0.25 ± 0.05), and high zeta potential (34 ± 0.5), and further experiments were carried out in this formulation.

**Fig. (3):**
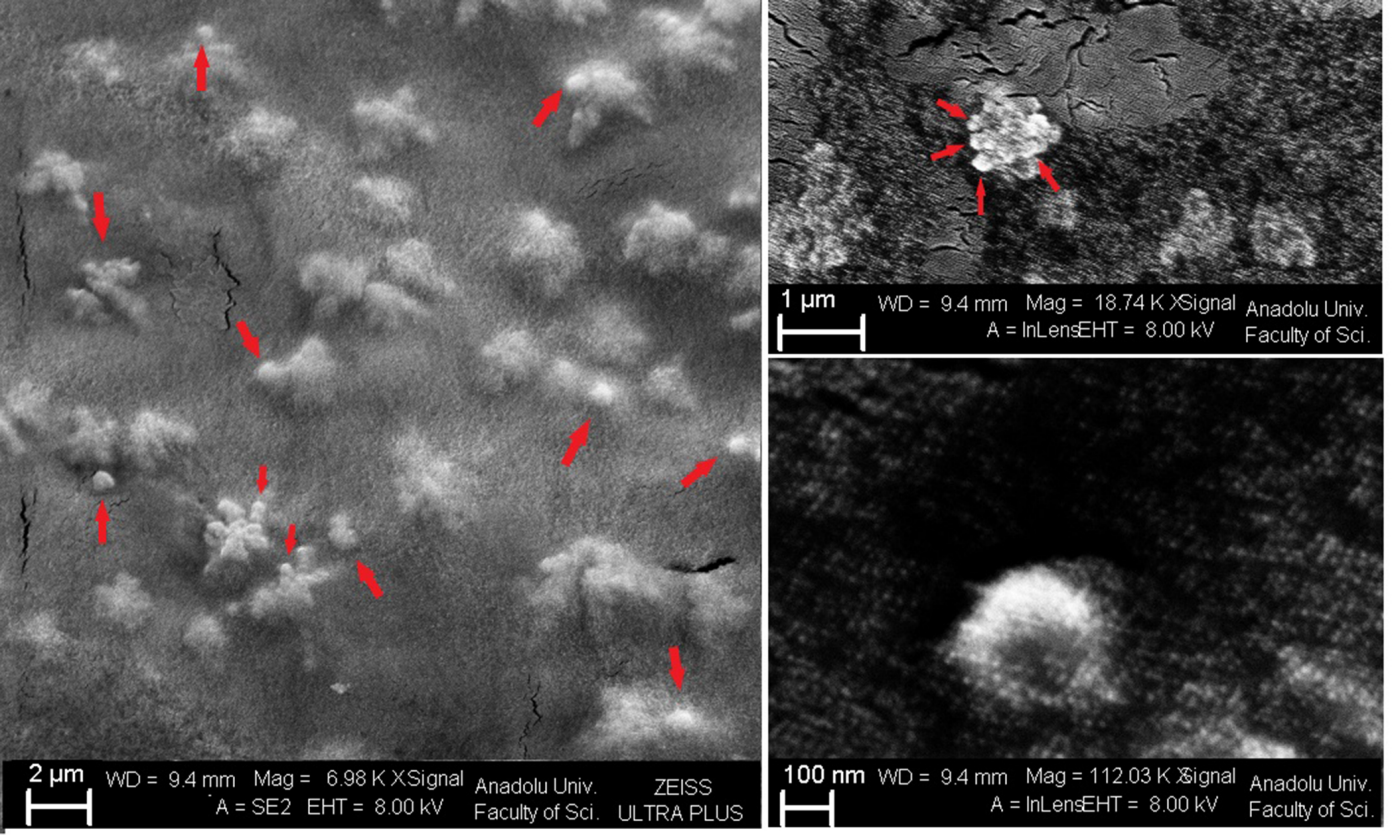
SEM images of K3-H4 formulation at 2 µm, 1 µm and 100 nm scaling.

Besides the particle size and surface charge of nanoparticles, nanoparticle morphology is also an important parameter affecting pharmacokinetics and cellular uptake. Therefore, it is crucial to examine the morphology of nanoparticles [44, 45]. It has been stated that the cell uptake of spherical nanoparticles is much higher than that of ellipsoidal nanoparticles, and chitosan nanoparticles also form spherical structures [46]. The SEM analysis of the K3-H4 formulation showed that nanoparticles had a spherical structure, and their particle sizes were 200-300 nm (Fig. **3**).

### 3.3 Gel retardation Studies

#### 3.3.1 Determination of HDL and Chitosan Nanoparticles Interactions

**Fig. (4):**
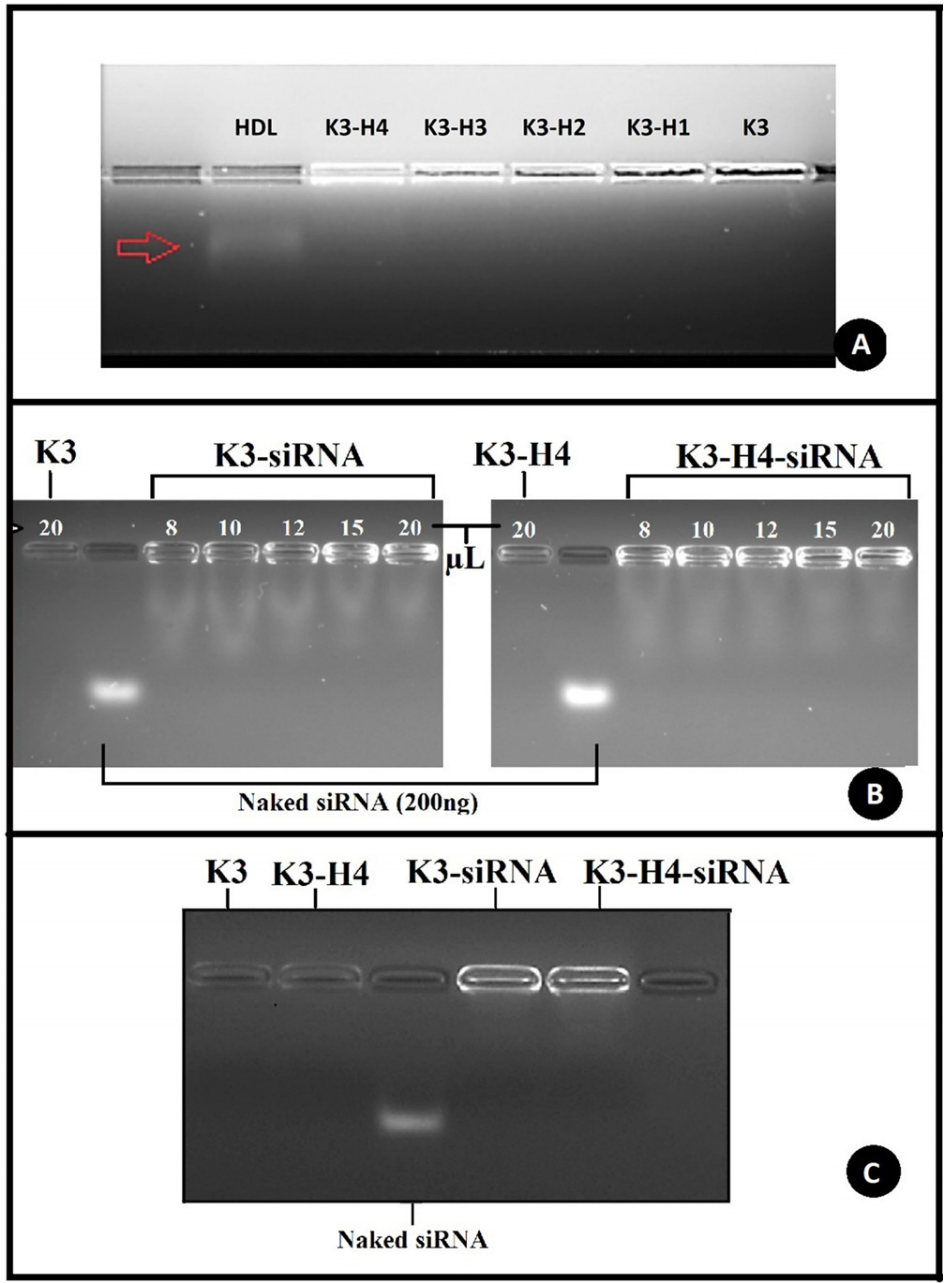
Agarose gel image of siRNA adsorbed formulations. (**A**) Agarose gel image of HDL, HDL-conjugated Formulations and K3 Formulation; (**B**) siRNA binding ability of K3 and K3-H4 formulations; (**C**) Capabilities of 20 mL K3 and K3-H4 formulations to bind 200 ng of siRNA.

It was reported that HDL does migrate during gel run towards the cathode because the surface charge of HDL particles is a low negative and does visible in the agarose gel that contains ethidium bromide (EtBr) using different staining [47]. This method is frequently used in biochemical studies to determine the location of HDL in agarose gel using staining methods, to separate HDL from other lipoproteins, and to quantify it [48, 49]. Since HDL was also seen on the gel in our study, different staining was not required. The main aim of this study was to determine whether negatively surface-charged HDL conjugated to chitosan nanoparticles and whether it would separate from the conjugated particles during electrophoresis. For this purpose, 120 µL of HDL solution and formulations were mixed separately with 30 µL of loading buffer and loaded onto 1% gel (Fig. **4A**).

The resulting gel image showed that the naked HDL migrated towards the cathode in the gel, while the HDLs contained in the formulation were stabilized in the wells. The radiation of the K3 formulation in the well showed that it belongs to chitosan, as in previous studies with chitosan [23]. Although there is the same amount of formulation for each well, the gradual decrease in radiation rates from K3-H4 to K3-H1 indicates that the radiation ratio depends on the number of HDLs contained in the formulation. In conclusion, the gel image proves that HDL has been successfully conjugated into the K3 formulation. The chitosan molecule dissolves in the acid medium, and the amine group (-NH2) is protonated. When the TPP solution is added, the negatively charged polyvalent TPP ions and the protonated amine groups on different or the same chitosan molecules can be crosslinked [38]. It is known that cross-linkers bind to the amino group of the lysine amino acid in the structure of proteins [50, 51]. It can be concluded that HDL and chitosan conjugation are provided by crosslinking between the lysine group of the ApoA-1 protein that surrounds HDL and chitosan.

#### 3.3.2 Interaction of siRNA with Formulations

The adsorption method, which is frequently used in gene delivery systems, was used to prepare nanoparticle-siRNA complexes. Complex formation between positively charged nanoparticles and negatively charged genes occurs *via* electrostatic interactions [23]. To determine the electrostatic interaction ratio between siRNA with K3 and K3-H4 formulations, 8 µl, 10 µl, 12 µl, 15 µl, and 20 µl samples of both formulations were taken into separate microtubes, and 200 ng of siRNA was added to each one. After incubation for electrostatic interaction, samples were loaded into the gel and imaged (Fig. **4B**).

The gel image showed smears that decreased with increasing formulation amounts. Since these smears were scattered behind the control siRNA band, they were not considered free siRNA in the formulation. Our previous study determined that genetic material could be separated from adsorbed particle surfaces by the electric current power during electrophoresis [52]. Electrophoresis was re-performed at 30 mV using the highest formulation dose (20µL) with 200 ng siRNA to confirm this prediction. The smear was not seen in the resulting gel image (Fig. **4C**). All results showed that K3 and K3-H4 were sufficient to bind the complete siRNA.

#### 3.3.3 Stability of siRNA in Serum

**Fig. (5):**
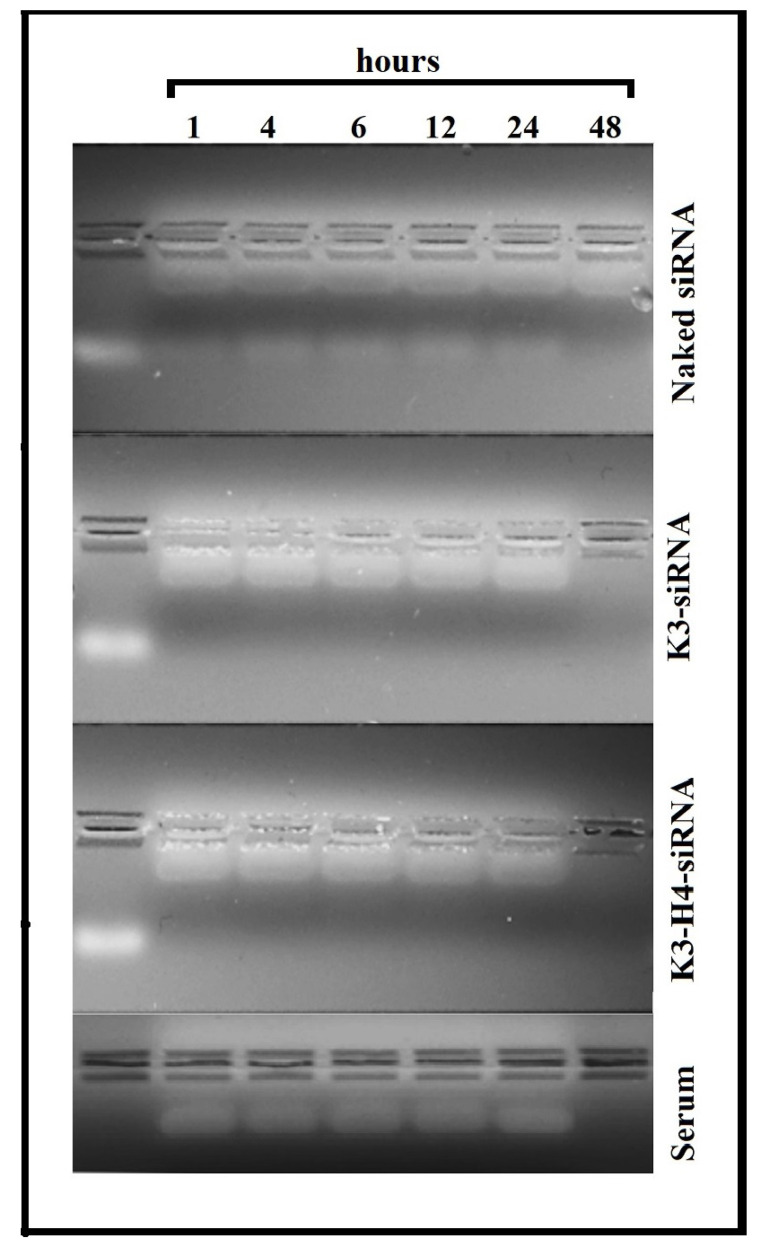
Gel image of siRNA, K3-siRNA and K3-H4-SiRNA following incubation with DMEM medium containing 10% FBS.

For the genetic material to exert its maximum effect in the cell, it must first be delivered into the cell by the carrier systems. These systems must protect the loaded genetic material from enzyme degradation in serum until the cell uptakes it [4, 53]. Naked siRNA, siRNA-K3, and siRNA-K3-H4 formulations were examined in the culture medium containing serum for different periods (1, 6, 12, 24, and 48 hours). The results showed that the naked siRNA was degraded entirely after 48 hours, while the K3 and K3-H4 formulations protected the siRNA from serum degradation (Fig. **5**). These results are consistent with studies on siRNA and siRNA/chitosan complexes [54, 55]. Serum was loaded on the gel. Then, it was subjected to electrophoresis to determine the origin of the bands observed at the bottom of the wells. The results revealed that these bands belong to the serum components.

### 3.4 Cellular Uptake

**Fig. (6):**
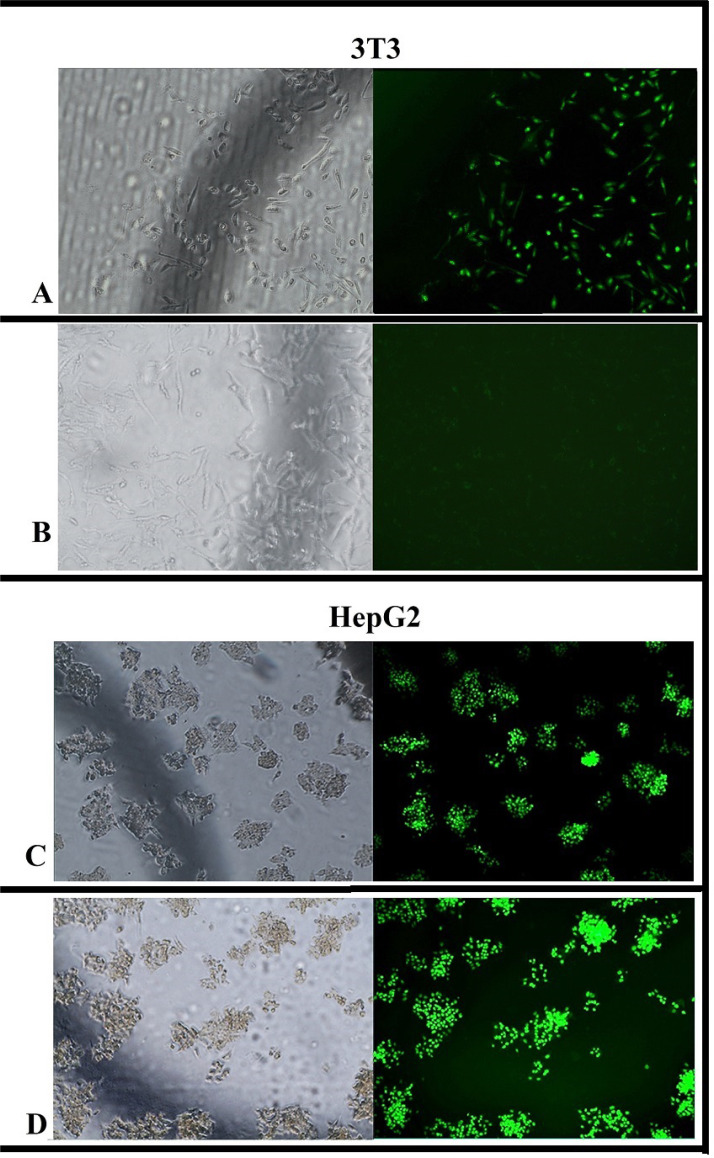
Transfection and cell culture studies of K3 and K3-H4 formulation in 3T3 and HepG2 cell lines. Transfection efficiency of K3 and K3-H4 formulation with FITC siRNA for 24 h. (**A**) Phase contrast and fluorescence imaging of 3T3 cells treated with K3-FITC siRNA, (**B**) 3T3 cells treated with K3-H4-FITC siRNA, (**C**) HepG2 cells treated with K3-FITC siRNA and (**D**) HepG2 cells treated with K3-H4-FITC siRNA.

Transfection studies were carried out with 200 ng FITC Conjugate siRNA adsorbed K3 and K3-H4 dispersions (20 µL) on HepG2 and 3T3 cell lines. After 24 hours of transfections study, cellular uptake percentages of each formulation by 3T3 and HepG2 cells were determined using plate reader software, and transfected cells were visualized with a fluorescence microscope (Fig. **6A**-**D**). According to these results, it was determined that the K3 formulation was taken up at a similar ratio by 3T3 cells (Fig. **6A**) and HepG2 cells (Fig. **6C**) (75 ± 0.85% and 76 ± 0.25%, respectively). Cationic particles can bind non-specifically to different cell types due to electrostatic interaction with the cell membrane, and this interaction significantly increases the internalization of them by cells [56, 57]. The observation of the similar high transfection rate of the K3-siRNA complex for both cells indicates that this complex can be internalized by cells non-specifically due to its positive charge [58]. The high rates of transfection indicate that at least one of the mechanisms of cellular uptake occurs for both cells, such as clathrin and/or caveolin-mediated endocytosis and internalization of the cell membrane due to the positive charge of the particles [40].

Uptake of the K3-H4-siRNA formulation by HepG2 cells was determined at a rate of 98 ± 0.15% (Fig. **6D**), while the plate reader software did not detect uptake by 3T3 cells. The absence of fluorescent spots in the image in Fig. (**6B**) also supports the result that the K3-H4-siRNA formulation was not transfected into 3T3 cells. This can be explained by considering the cell-specific lipid transport mechanism of HDL after binding to SR-B1. Although it is known that HDL can affect the cellular signal by binding to cell surface receptors, the complexity of its metabolism is still not fully understood. On the other hand, It has been known that HDL binds to different cell types with varying degrees of specificity. After HDL binds to the receptor it has an affinity for, the lipids it carries are transferred to the cells in two ways, depending on the characteristics of the cells. The first way is to transfer lipids to cells without particle catabolism, predominantly by selective lipid uptake, while the second way is to uptake the whole HDL particle in the cells through endocytosis and discharge lipids [59]. The liver is where the biosynthesis of ApoA-1 and HDL begins and is where returned cholesterol-enriched HDL degradation takes place. SR-BI transfers cholesterol to hepatocytes by selective lipid uptake and mediates the movement of cholesterol ester from the hydrophobic HDL core to the cell. In addition, SR-BI and other low-affinity HDL binding sites help to mediate HDL holo-particle uptake. After endocytosis, HDL releases cholesterol into hepatocytes and is used for the formation of new lipoproteins or the secretion of bile and bile acids [15, 59, 60]. Transfection results show that HepG2 cells, human hepatocyte cells [61, 62], uptake the HDL-containing K3-H4-siRNA formulation by endocytosis mechanism associated with their tendency to internalize HDL. The absence of transfection for 3T3 cells suggested that this cell type uses the selective lipid uptake pathway rather than endocytosis. The reason why the K3-H4-siRNA formulation could not be uptaken by the cell was interpreted as HDL, which does not contain the lipid demanded by the cell, is released from SR-B1 for recirculation although its interaction with SR-B1 on the cell membrane. In addition, overexpression of the SR-B1 receptor on the membrane of malignant cells may be increased the number of HDL-conjugated particles to bind to the cell membrane and the rate of their uptake into the cell compared to 3T3 cells.

The tendency of HepG2 and 3T3 cells to utilize HDL by different cellular mechanisms and the high affinity of HDL to cells overexpressing SR-B1 indicated that the siRNA delivery system K3-H4-siRNA formulation could be highly uptake by malignant hepatocyte cells.

#### 3.4.1 Cytotoxicity of Formulations

Fig. (**7**) shows results obtained after 24 and 48 h treatment of 3T3 and HepG2 cells with all formulations and naked siRNA. According to the results, it was found that all formulations and naked siRNA for 24 hours were not cytotoxic for each cell type. The fact that naked siRNA did not affect cell viability in both periods indicates that siRNA was not taken into the cell in the first 24 hours and degraded enzymatically in a cell culture medium containing serum after 48 hours. The highest dose of K3-siRNA complex reduced cell viability from 128.83 ± 1.45 to 72.35 ± 0.72% at 48 hours; this indicates that Bcl 2-siRNA effectively silences the Bcl 2 gene after uptake into 3T3 cells. Based on the knowledge that the K3-siRNA complex is uptake by 3T3 cells at a rate of 75% ± 0.85%, this result shows that Bcl 2-siRNA does not offer its efficacy in the first 24 hours but begins to act after 24 hours. It is known that overexpression of Bcl-2 is higher in cancer cells than in healthy cells. Considering that the Bcl2 is not overexpressed by 3T3 healthy cells, it is a predictable situation that the K3-siRNA formulation would show low toxicity [63]. Although it was determined that the K3-H4-siRNA complex did not uptake into 3T3 cells within 24 hours, the highest dose of this complex reduced cell viability from 99.50 ± 2.03% to 83.15 ± 1.52%. It has been reported that the strong electrostatic interaction between the cationic nanoparticles and the phosphate groups of the cell membrane leads to an increase in the membrane surface tension, resulting in the formation of pores [40, 64]. This result can be interpreted as the formation of pores on the cell membrane due to the electrostatic interaction between the cell membrane and the K3-H4-siRNA complex that interacted with SR-B1 but cannot be taken up by the cell.

For HepG2 cells, dose-dependent cytotoxicity was determined in all formulations except naked siRNA after 48 hours. The decrease in HepG2 cell viability observed after 48 hours at all doses of K3 and K3-H4 formulations was associated with the antitumor mechanism of chitosan nanoparticles. Due to their positive character and apoptosis-inducing activities, the antitumor mechanism of chitosan nanoparticles depends on their membrane-disrupting properties [64]. Comparing the cytotoxicities of K3-siRNA and K3-H4-siRNA revealed statistical similarities (*p* > 0.05) between both formulations at concentrations in the 8-15 µm range. When the transfection effects of these two formulations were compared, the K3-H4-siRNA formulation was expected to be more cytotoxic than the K3-siRNA formulation. The statistically similar cytotoxic efficacy of K3-siRNA to K3-H4-siRNA was associated with the difference in size and zeta potential between particles. It has been reported that the physiochemical properties of chitosan nanoparticles, such as particle size and zeta potential, can significantly change their antitumor activities [64]. The smaller particle size of the K3-siRNA formulation than the K3-H4-siRNA formulation may result in the binding of more nanoparticles per cell, resulting in increased cell wall disruption and decreased cellular viability.

The K3-H4-siRNA formulation was the most toxic formulation when all concentrations were evaluated. Among all formulations, only the highest dose of K3-h4-siRNA was found to reduce cell viability below 50% (IC_50_) (42.01±1.83%). This result was already expected based on the transfection results. The possible explanation is that the introduction of HDL conjugate promotes the cellular uptake of siRNA-loaded nanoparticles by HepG2 cells *via* SR-B1 receptor-mediated endocytosis, leading to higher cytotoxicity.

## CONCLUSION

In conclusion, we have demonstrated that HDL-conjugated chitosan nanoparticles could be used as a siRNA delivery system for targeting the SR-B1 receptor. After it was proved that HDL obtained from the blood taken from volunteers contains ApoA-1 protein with affinity for the SR-B1 receptor, electrophoresis methods showed that HDL conjugated to chitosan particles occurred. The selective higher cellular uptake of HDL-conjugated nanoparticles by SR-B1 overexpressing cells was proven by comparing this system with transfection studies of chitosan particles without HDL by SR-B1 overexpressing cells and healthy cells. Cytotoxicity results in HepG2 cells showed that Bcl-2 siRNA blocked the target gene and induced apoptosis in HepG2 cells. In summary, by conjugating HDL, the chitosan-based siRNA delivery systems can target malignant cells that consistently overexpress SR-B1 receptors, such as HepG2.

Overall, our study supports the HDL/chitosan/siRNA system can be specifically targeted SR-B1 overexpressed cancer cells and be used as a drug delivery system in the targeted therapy of liver cancer. Thus, it is thought that this system would be able to provide the local release of genetic material at the target site, the liver, and reduce the dose and dose-related toxicity. Even if this siRNA delivery system is uptake by healthy hepatocytes *via* receptor-mediated endocytosis, it may be less toxic for them compared to malignant hepatocytes. Since hepatocytes do not overexpress Bcl-2 and SR-B1, it has been concluded the rate of Bcl-2 siRNA-associated toxicity and the ratio of the particle binding to SR-B1, would be low compared to malignant hepatocytes, as determined to 3T3 cells. If this study is developed, it may be used to create a synergistic effect with chemotherapy and radiotherapy against cells that are resistant to these treatments. It is also possible to use these systems to provide more active treatment with combinations of genetic material targeting different cellular functions.

## Figures and Tables

**Fig. (1):**
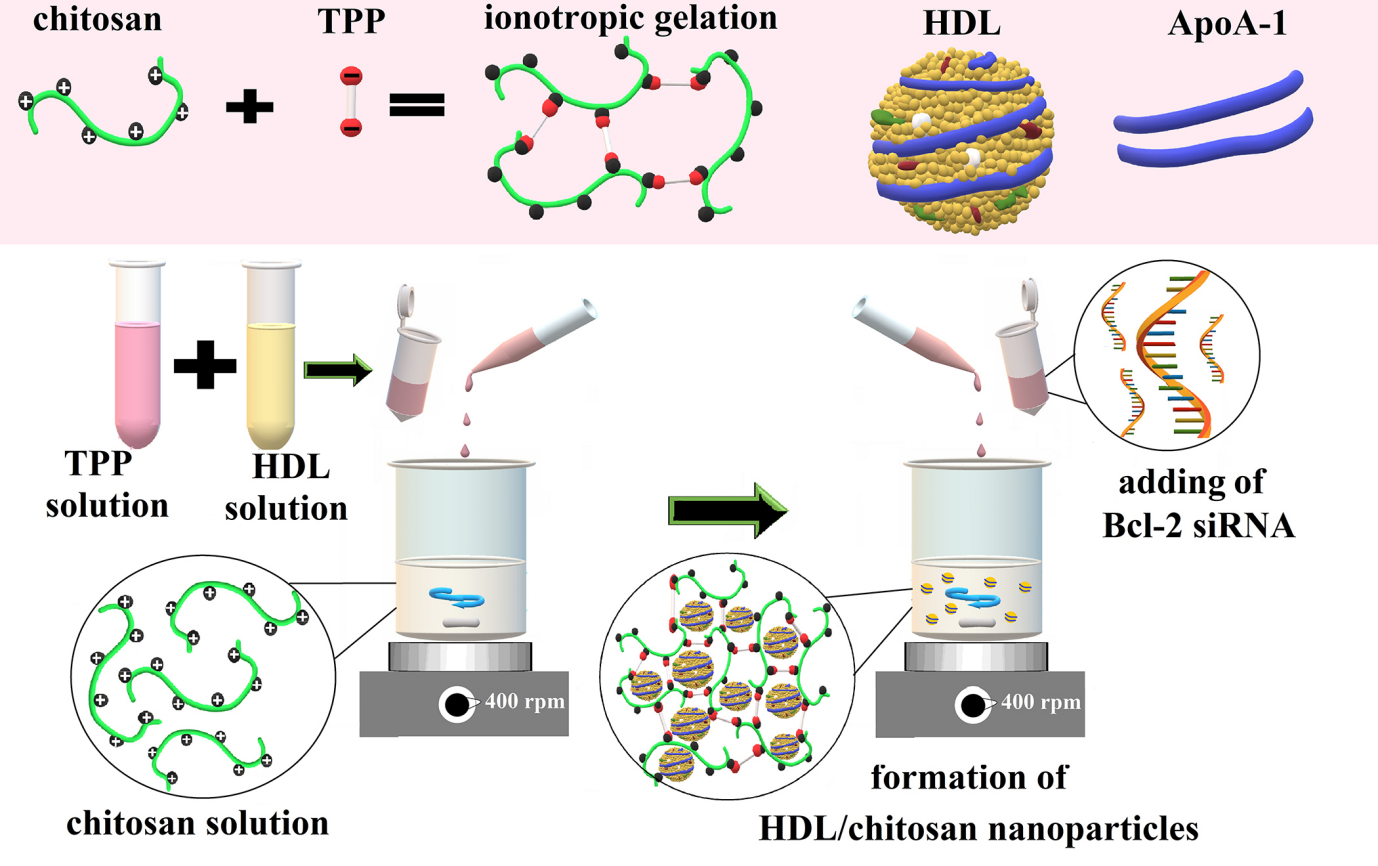
Schematic illustration of the preparation of HDL/Chitosan/siRNA formulations.

**Fig. (2):**
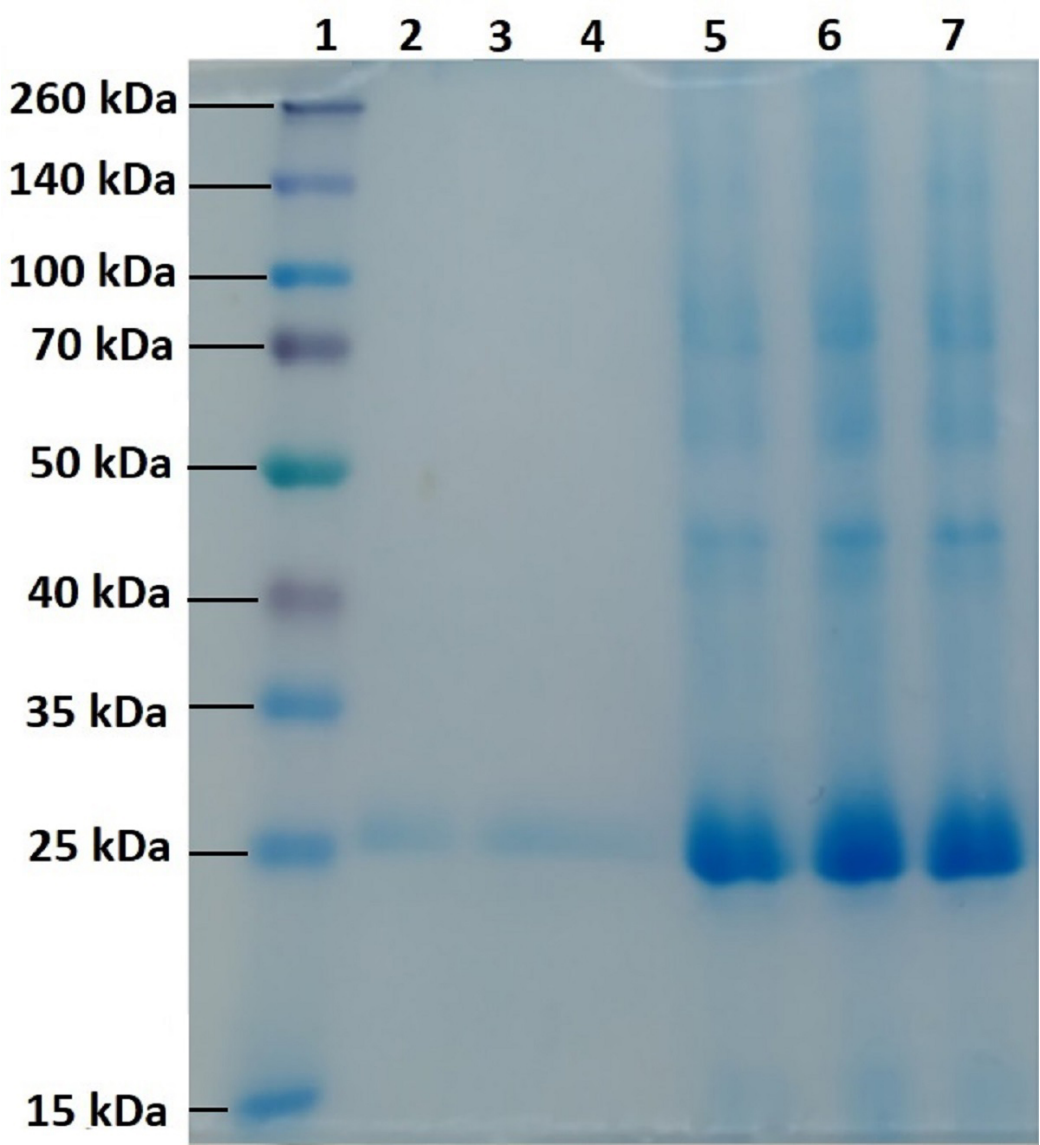
SDS-PAGE image after isolation of ApoA-1 protein. Line 1: Prestained Protein Ladder; Line 2-4: ApoA-1 protein; Line 5-7: HDL.

**Fig. (7):**
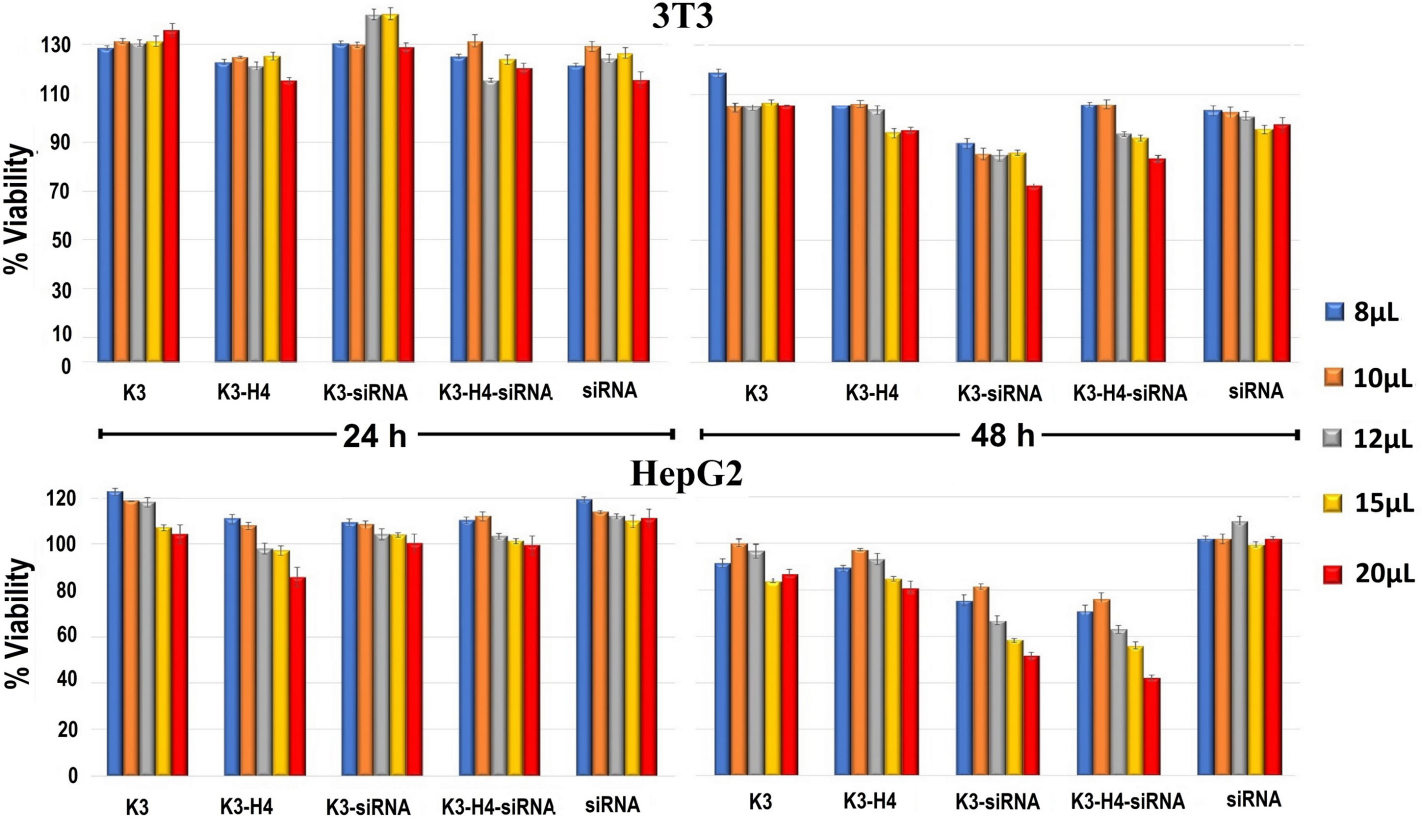
Viability of 3T3 and HepG2 cells after being treated with naked Bcl 2-siRNA, K3, K3-H4 formulation and its complex with Bcl 2-siRNA. Each formulation was tested in eight replicates for 24 and 48 h. For Bcl 2-siRNA, the quantities in the formulations (80, 100, 120, 150 and 200 ng/100 μL) were used.

**Table 1:** The codes, compositions, average diameters, particle size distributions and zeta potentials of the nanoparticles.

Codes	HDL	AD (nm) ± SE	PDI ± SE	ZP (mV) ± SE
**K3**	*-*	*170 ± 10*	*0.285 ± 0.05*	*36 ± 0.1*
**K3-H1**	500 µl	625 ± 75	0.757 ± 0.2	27 ± 1.0
**K3-H2**	250µl	460 ± 17	0.453 ± 0.05	29 ± 0.5
**K3-H3**	100µl	376 ± 26	0.406 ± 0.05	32 ± 0.2
**K3-H4**	75µl	240 ± 10	0.251 ± 0.05	34 ± 0.5

**Note:** % Chitosan solution (w/v) /amount (mL)= 0,5/2,24; mass ratio of chitosan/TPP= 4/1; concentration of HDL Solution: 5.7 mg/mL; **Abbreviations:** AD = Average diameter; PDI = Polydispersity index; ZP = Zeta potential, and SE = Standard error.

## Data Availability

This study is a master's thesis written in Turkish by Rasim Masimov and can be accessed from the Turkish National Thesis Center (https://tez.yok.gov.tr/UlusalTezMerkezi/) with the number 599070 upon request.

## LIST OF ABBREVIATIONS

Apoa-1: Apolipoprotein AI
ATP-ABCA1: Binding Cassette Transporter AI
DMEM: Dulbecco's Modified Eagle's Medium
EDTA: Ethylenediamine Tetraacetic Acid
EtBr: Ethidium Bromide
FBS: Fetal Bovine Serum
FITC: Fluorescein İsothiocyanate
HDL: High-Density Lipoprotein
LCAT: Lecithin/Cholesterol Acyltransferase
MTT: 3-(4,5-dimethylthiazol-2-yl)-2,5-diphenyltetrazolium bromide
PDI: Polydispersity İndex
PEC: Polyelectrolyte Complex
RCT: Reverse Cholesterol Transport
RES: Reticuloendothelial System
SEM: Scanning Electron Microscope
SR-B1: Scavenger Receptor Class B Type 1
TBE: Tris/Borate/EDTA
TPP: Tripolyphosphate
ZP: Zeta Potential
